# Estimation of extravascular lung water using the transpulmonary ultrasound dilution (TPUD) method: a validation study in neonatal lambs

**DOI:** 10.1007/s10877-015-9803-7

**Published:** 2015-11-12

**Authors:** S. L. Vrancken, A. Nusmeier, J. C. Hopman, K. D. Liem, J. G. van der Hoeven, J. Lemson, A. F. van Heijst, W. P. de Boode

**Affiliations:** 1 Department of Pediatrics - Neonatology, Radboud University Medical Center, Internal Postal Code 804, P.O. Box 9101, 6500 HB Nijmegen, The Netherlands; 2 Department of Intensive Care Medicine, Radboud University Medical Center, Nijmegen, The Netherlands; 3 Department of Radiology and Nuclear Medicine, Radboud University Medical Center, Nijmegen, The Netherlands

**Keywords:** Cardiac output, Extravascular lung water, Monitoring, Children, Neonate, Dilution technique, Indicator, Hemodynamics

## Abstract

Increased extravascular lung water (EVLW) may contribute to respiratory failure in neonates. Accurate measurement of EVLW in these patients is limited due to the lack of bedside methods. The aim of this pilot study was to investigate the reliability of the transpulmonary ultrasound dilution (TPUD) technique as a possible method for estimating EVLW in a neonatal animal model. Pulmonary edema was induced in 11 lambs by repeated surfactant lavages. In between the lavages, EVLW indexed by bodyweight was estimated by TPUD (EVLWI_tpud_) and transpulmonary dye dilution (EVLWI_tpdd_) (n = 22). Final EVLWI_tpud_ measurements were also compared with EVLWI estimations by gold standard post mortem gravimetry (EVLWI_grav_) (n = 6). EVLWI was also measured in two additional lambs without pulmonary edema. Bland–Altman plots showed a mean bias between EVLWI_tpud_ and EVLWI_tpdd_ of −3.4 mL/kg (LOA ± 25.8 mL/kg) and between EVLWI_tpud_ and EVLWI_grav_ of 1.7 mL/kg (LOA ± 8.3 mL/kg). The percentage errors were 109 and 43 % respectively. The correlation between changes in EVLW measured by TPUD and TPDD was r^2^ = 0.22. Agreement between EVLWI measurements by TPUD and TPDD was low. Trending ability to detect changes between these two methods in EVLWI was questionable. The accuracy of EVLWI_tpud_ was good compared to the gold standard gravimetric method but the TPUD lacked precision in its current prototype. Based on these limited data, we believe that TPUD has potential for future use to estimate EVLW after adaptation of the algorithm. Larger studies are needed to support our findings.

## Introduction

Extravascular lung water (EVLW) is defined by the fluid within the lung but outside the vascular compartment and is the total of interstitial fluid, alveolar fluid, extravasated plasma, intracellular water, lymphatic fluid and surfactant [1]. Respiratory failure in critically ill newborns is mainly due to respiratory distress syndrome (RDS), meconium aspiration and pneumonia, conditions that are often associated with increased capillary permeability and EVLW. Complex systemic inflammatory reactions as seen during sepsis, on cardiopulmonary bypass or extracorporeal membrane oxygenation, can also affect the microvascular permeability in neonates resulting in pulmonary fluid overload with further impairment of pulmonary gas exchange. In critically ill adults and children, the amount of EVLW is a good predictor of mortality [2–5] and therapy guided by EVLW measurements seems to have beneficial effects on outcome [6, 7]. Only a few studies investigated the role of EVLW in newborn infants as a prognostic factor for ventilator dependency and outcome [8, 9]. Still, measurement of extravascular lung water could enable the neonatologist to optimize fluid management in order to improve the pulmonary function.

The gold standard and reference method to measure EVLW is post-mortem gravimetry, a method which use is mainly restricted to experimental settings. Measurement of the dry and wet weight of the removed lungs enables the calculation of EVLW [10–12]. Transpulmonary indicator dilution methods are used in adult and pediatric intensive care to estimate EVLW. The transpulmonary double dilution (TPDD) technology using ice-cold indocyanine green (ICG) is regarded the clinical gold standard [13–15], but the TPDD system is not longer commercially available. Currently, the transpulmonary single thermal indicator dilution (TPTD) technique using ice-cold saline is increasingly used bedside for the estimation of EVLW [16–18]. However, this method cannot be used in newborn infants (<3.5 kg) due to size limitations of the dedicated catheter. The transpulmonary ultrasound dilution (TPUD) technology utilizes pre-existing central venous and arterial lines and is applicable for neonatal cardiac output (CO) measurement, even in the presence of (extra-)cardiac shunts [19, 20]. TPUD is based on changes in ultrasound velocity in arterial blood after central venous injection of isotonic saline at body temperature [21]. When used as a double indicator method with injection of ice-cold isotonic saline, EVLW can be calculated using the difference in mean transit time (MTt) between the diffusible (heat) and non-diffusable (saline) indicator.

The aim of this study was (1) to validate EVLW measurements using TPUD against gravimetry over a wide range of lung water values in a neonatal animal model and (2) to compare sequential EVLW measurements of TPUD and TPDD to validate the possibility of tracking changes in EVLW measured by TPUD in the same model.

## Materials and methods

### General

This experiment was performed in accordance with Dutch national legislation concerning guidelines for the care and use of laboratory animals, approved by the Ethical Committee on Animal Research of the Radboud University Nijmegen (RU-DEC #2010-034 and RU-DEC #2010-034A1) and performed in thirteen lambs under general anesthesia. They were premedicated with an intramuscular injection of ketamine (10 mg/kg), atropine (0.03 mg/kg) and midazolam (0.2 mg/kg) and intravenous administration of propofol (2 mg/kg). General anesthesia was maintained with inhalation of isoflurane (0.5–2.0 vol%), and intravenous administration of sufentanyl (15–25 μg/kg/h), midazolam (0.2 mg/kg/h) and pancuronium (0.02 mg/kg/h after a loading dose of 0.05 mg/kg). Anaesthetics were adapted if the depth of anesthesia—repeatedly assessed by pain stimuli and clinical parameters such as heart rate, spontaneous ventilation, and arterial blood pressure—was insufficient. The lambs were orotracheally intubated with a cuffed endotracheal tube (inner diameter 4–6 mm; Kruse, Marslev, Denmark) and their lungs were mechanically ventilated in a pressure control mode [Datex Ohmeda Excel 210 SE anesthesia machine (GE Healtcare, Waukesha, Wisconsin, USA)] with tidal volumes of approximately 8–10 mL/kg and inspiratory-to-expiratory ratio of 1:2. Normocapnia, estimated by capnography with the CO2SMO Plus Respiratory Profile Monitor (Model 8100; Respironics, Pittsburgh, PA), was realized by adjusting the minute volume ventilation to maintain an end-tidal CO_2_ between 30 and 41 torr (4.0–5.5 kPa). In case of decreased oxygenation positive end-expiratory pressure (PEEP) and/or the FiO_2_ were adjusted to maintain the measured arterial oxygen saturation above 95 %. A servo-controlled heating mattress and a heating radiator were used to maintain a rectal temperature between 38 and 39 °C. The animals were euthanized at the end of the experiment by a lethal dose of pentobarbital (150 mg/kg intravenously).

### Instrumentation

Immediately after induction of anesthesia, several intravascular catheters were inserted by surgical cut-down:

1. A thermal-dye-dilution probe (Pulsiocath PV2023, 3F; Pulsion) equipped with a thermistor for detection of changes in blood temperature and a fiber optic to detect changes in ICG concentrations in the blood was placed in the lower abdominal aorta via the right femoral artery,
2. In the contralateral femoral artery, an arterial catheter (umbilical vessel catheter 5 Ch/35 cm/1.7 mm, Argyle™, Tyco Healthcare/Kendall Ireland Limited, Tullamore, Ireland) was introduced—also positioned in the abdominal aorta—and connected with the arterial limb of the extracorporeal circuit for TPUD measurements (see later). This catheter was also used for continuous blood pressure monitoring and blood sampling,
3. A central venous catheter (5F, 2 lumen, 13 cm; Arrow International, Reading, PA) was inserted in the ipsilateral femoral vein with regard to the thermal-dye-dilution probe, with the tip located near de right atrium for the injection of ice-cold indocyanine green in order to avoid a cross talking phenomena [22] and
4. A double-lumen central venous catheter (16 G/16 cm/1.7 cm, Arrow, Arrow International, Reading, PA, USA) was inserted via the right jugular vein with the position of the tip in the superior vena cava. One of the lumina of this central venous catheter was connected to the venous limb of the AV-loop for TPUD measurement. The other lumen was used for administration of fluids and medication.

### Induction of lung injury

In 11 lambs, lung injury and pulmonary edema was stepwise induced using a surfactant depletion model as described by Lachmann et al. [23]. Prior to the lavages, the lambs were pre-oxygenated with FiO_2_ 1.0. Repetitive lung lavages were performed by instilling 10–30 mL/kg/lavage isotonic saline (37 °C). After a 2–3 min period the saline was drained by gravity and subsequently suctioned with an endotracheal suction catheter (10 Ch/60 cm, Mülly, Unomedical A/S, Denmark). Ventilator settings (PEEP and minute volume ventilation) were adjusted in order to maintain arterial oxygen saturation and end-tidal CO_2_ within the target ranges. Lavages were repeated until a PaO_2_ < 100 torr (<13.3 kPa) was achieved at a FiO_2_ of 1.0 [24]. The lambs remained in supine position during the experiment to obtain a bilateral washout. In two additional lambs, only baseline measurements were performed without surfactant wash out to include also EVLWgrav measurements of minimal injured lungs before they were euthanized.

Oxygenation index (OI) was used as an indicator to assess the severity of the induced lung injury during the experiment and calculated as (mPAW × FiO_2_)/PaO_2_ with mPAW the mean airway pressure (cm H_2_O), FiO_2_ the inspired oxygen (%) and PaO_2_ the partial oxygen tension (torr). mPAW was calculated as [(PIP × T_insp_) + (PEEP × T_exp_)]/T_insp_ + T_exp_ where PIP is the peak inspiratory pressure (cm H_2_O), T_insp_ the inspiratory time (s), PEEP the positive end-expiratory pressure (cm H_2_O), and T_exp_ the expiratory time (s). A higher OI was indicative of more severe lung injury.

### Transpulmonary ultrasound dilution

TPUD uses isotonic saline at body temperature as an indicator to estimate cardiac output and is based on the principle that ultrasound velocity differs between blood (1560–1590 m/s) and isotonic saline (1533 m/s) [21]. An extracorporeal arterio-venous loop, equipped with ultrasound sensors on both venous (for calculating the exact amount and onset of injected indicator) and arterial site (for detection of changes in ultrasound dilution), is connected between the indwelling arterial and central venous catheters. Blood flows through this loop only during measurement procedures and is controlled by a peristaltic pump to prevent stasis of blood and provide stable blood flow. To measure EVLW the TPUD was converted into a double indicator method using ice-cold (<10 °C) isotonic saline. An additional thermistor was placed on the arterial side of the loop for detection of changes in blood temperature. Isotonic saline is considered to be non-diffusible and travels only in the intravascular space, including the heart, lungs, and blood vessels during its first pass as a consequence of an absent concentration gradient across lung capillaries [25]. The second indicator (heat) is diffusible and travels in both the intravascular space and the extravascular lung space. The difference between the mean transit time (MTt) of the diffusible indicator (measured by thermistor) and the non-diffusible indicator (measured by ultrasound sensor) was used to calculate EVLW.

Calculation of lung water uses the following equation:

1 $${\text{EVLW}} = {\text{CO}} \times \left( {{\text{MTt}}_{\text{ther}} {-}{\text{MTt}}_{\text{UD}} } \right)$$

where CO is the cardiac output, MTt_ther_ is the mean transit time of the thermal dilution curve recorded by the thermistor and MTt_UD_ is the mean transit time of the ultrasound dilution curve recorded by the ultrasound sensor.

Corrections must be made for (1) the increase in ultrasound velocity during passage in the plastic tubing due to blood cooling from cold saline, (2) the distribution of temperature not only in the blood but also in the tubing walls during its passage (∆t_tube_) and (3) any delay of temperature transfer to the thermal sensor when the cooled blood arrives to the location of the thermistor (∆t_ts_).

Equation 1 is therefore revised as follows:

2 $${\text{EVLW}} = {\text{CO}} \times \left[ {\left( {{\text{MTt}}_{\text{therm}} {-}\Delta {\text{t}}_{\text{tube}} {-}\Delta {\text{t}}_{\text{ts}} } \right){-}\left( {{\text{MTt}}_{\text{UD}} +\Delta {\text{t}}_{\text{pl}} } \right)} \right]$$

where ∆t_tube_, time delay related to heat exchange within AV loop tubing; ∆t_ts_, time delay related to inertia of thermistor due to plastic surroundings and ∆t_pl_, time accounting for the influence of ultrasound velocity changes in plastic tubing.

Each EVLW measurement session consisted of two ice-cold (<10 °C) isotonic saline injections (1 mL/kg). EVLW was determined by the average of two consecutive EVLW measurements by TPUD and indexed by bodyweight (EVLWI_tpud_). Specially designed software (CO-status^®^, Transonic Systems Inc., Ithaca, NY, USA) was used to calculate cardiac output, hemodynamic volumes and EVLW. Values were displayed on the monitor.

### Transpulmonary double indicator dilution (see “Appendix 1”)

TPDD measurements were performed by rapid injection of 5-mL ice-cold (<10 °C) ICG (1 mg/mL in glucose 5 %) into the femoral venous catheter. Changes in both temperature and ICG concentration were detected by the thermal-dye-dilution probe connected to a COLD monitor (Pulsion Medical Systems, Germany). Cardiac output, blood volumes, and EVLW were calculated from the analysis of the dilution curves and measurement of the mean transit time and downslope time [26, 27]. Before a series of measurements was performed, the central venous catheter was flushed with 1–2 mL of ice-cold saline. Each dilution curve was visually inspected for artefacts or other signs of inadequate measurement. EVLW was determined by the average of three consecutive EVLWI measurements by TPDD and indexed by bodyweight (EVLWI_tpdd_). Unfortunately, in the last three animal experiments, the COLD system was defective.

### Gravimetric technique (see “Appendix 2”)

50-mL of central venous blood was collected immediately prior to the euthanasia. Subsequently, the lungs were removed after median sternotomy and drained passively. The gross weight of each lung, with its residual blood, was determined. The lungs were homogenized with an equal weight of water to induce hemolysis using a commercial blender and homogenizer. Half of the homogenate was used to determine its wet/dry weight and the other half was centrifuged at 6000 rpm for 1 h at 4 °C to separate a clear supernatant. Hematocrit from whole blood and hemoglobin from whole blood and lung supernatant were measured. The wet and dry weights of the samples of blood, homogenate, and supernatant were determined before respectively after 4 days of incubation in a heat chamber at 70 °C. Post mortem EVLWI was calculated by the formulas as previously described (see “Appendix 1”) and also indexed by bodyweight (EVLWI_grav_) [10–12].

### Experimental protocol

After a stabilization period of 15 min following instrumentation the study protocol was started. Sessions of EVLWI_tpud_ measurements and blood gas analysis were performed at baseline and after a postlavage stabilization period of 15 min following 2 consecutive lavages. At baseline, halfway and at the end of the experiment EVLWI_tpdd_ was also estimated. In all lambs, postmortem measurements of extravascular lung water were performed by gravimetry. The median time interval between the last EVLW measurement and the euthanasia was 40 (IQR 4) minutes with a range between 15 and 60 min. A blood transfusion was administered if the hemoglobin concentration was less than 5.6 g/dL. Inotropic support (dobutamine or epinephrine) was initiated in case of circulatory instability (hypotension and/or low cardiac output) due to hypoxia.

### Statistics

The cardiac output values were indexed by bodyweight (mL/kg/min). Variables were summarized as frequencies for categorical data or means, and standard deviations (SD) for continuous normally distributed variables. A Mann-Witney test was used for comparison between initial and final oxygenation indexes and PEEP levels in the lambs that underwent lung lavages. Data from the different EVLWI measurements were compared using the method described by Bland and Altman [28]. The bias was defined as the difference between EVLWI_tpud_ and EVLWI measured by the reference method (EVLWI_tpdd_/grav) or the difference between EVLWI_tpdd_ and EVLWI_grav_. The bias was plotted against the mean EVLWI of both methods [(EVLWI_tpud_ + EVLWI_tpdd_/grav)/2 or (EVLWI_tpdd_ + EVLWI_grav_)/2]. The limits of agreement (LOA) were calculated by multiplying the SD of the bias with 1.96. The percentage error (PE) was calculated as 100 × [(1.96 × SD of the bias)/mean EVLWI(tpdd/grav)] [29]. We did not correct for (unequal) repeated measurements as the number of paired measurements per subject was small (1–5) and less than the total number of subjects (13), allowing pooling of the data [30]. Comparison of bias of EVLWI between TPUD and the two reference methods and final PEEP, final oxygenation index, mean EVLWI and CO were analyzed by Spearman coefficient of rank correlation. Changes in EVLWI measurements by TPUD and TPDD were calculated by subtracting the consecutive EVLWI measurements. Comparison of these changes in EVLWI was analyzed by Spearman coefficient of rank correlation. A *p* value <0.05 was considered statistically significant. SPSS 20 for Windows^®^ (SPSS Inc., Chicago, USA) was used for statistical analysis.

## Results

**Fig. 1:**
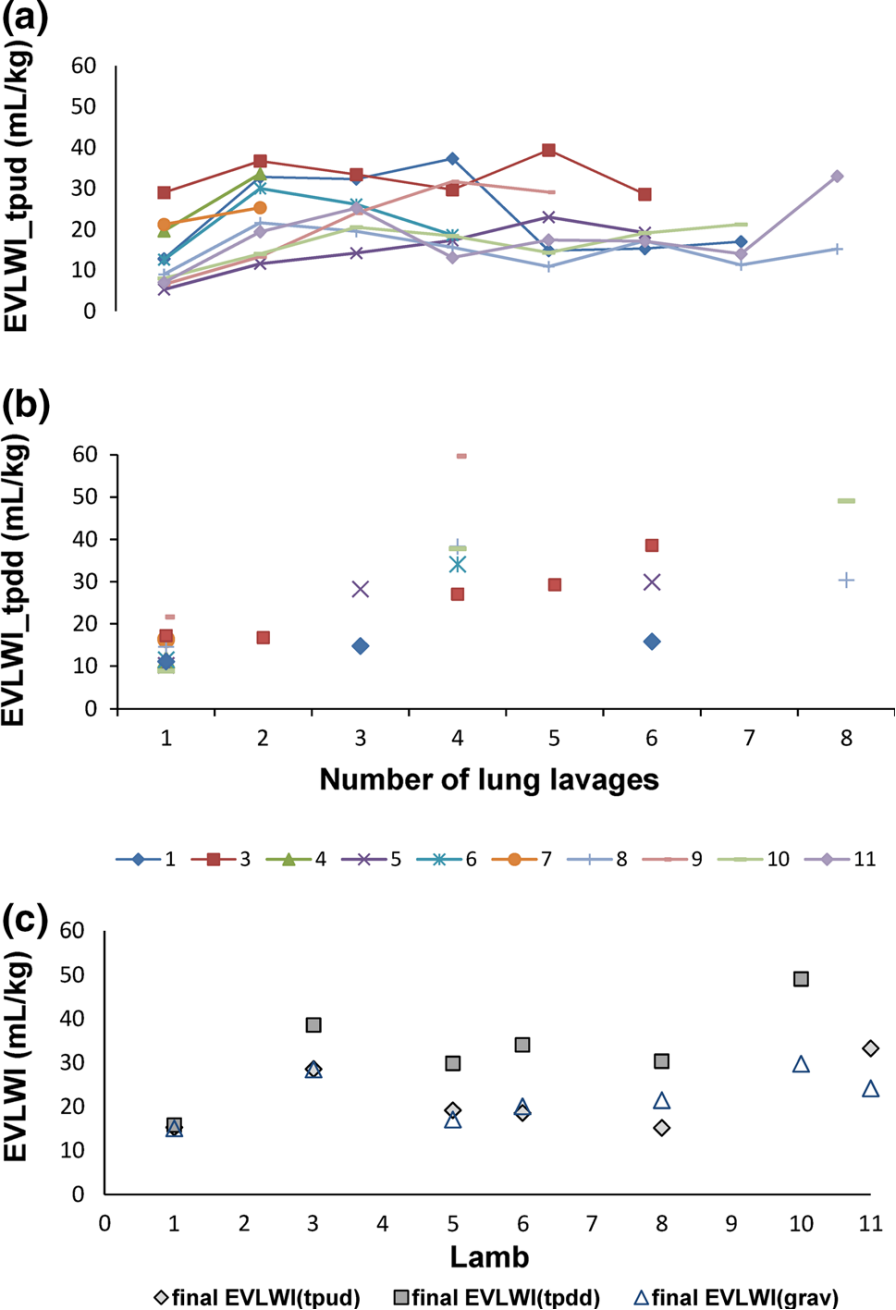
Results of the extravascular lung water index (EVLWI) measured by transpulmonary ultrasound dilution (TPUD) (**a**) and transpulmonary double dilution (TPDD) (**b**) during the experiment and of the final EVLWI measurements by the three different methods (**c**)

**Fig. 2:**
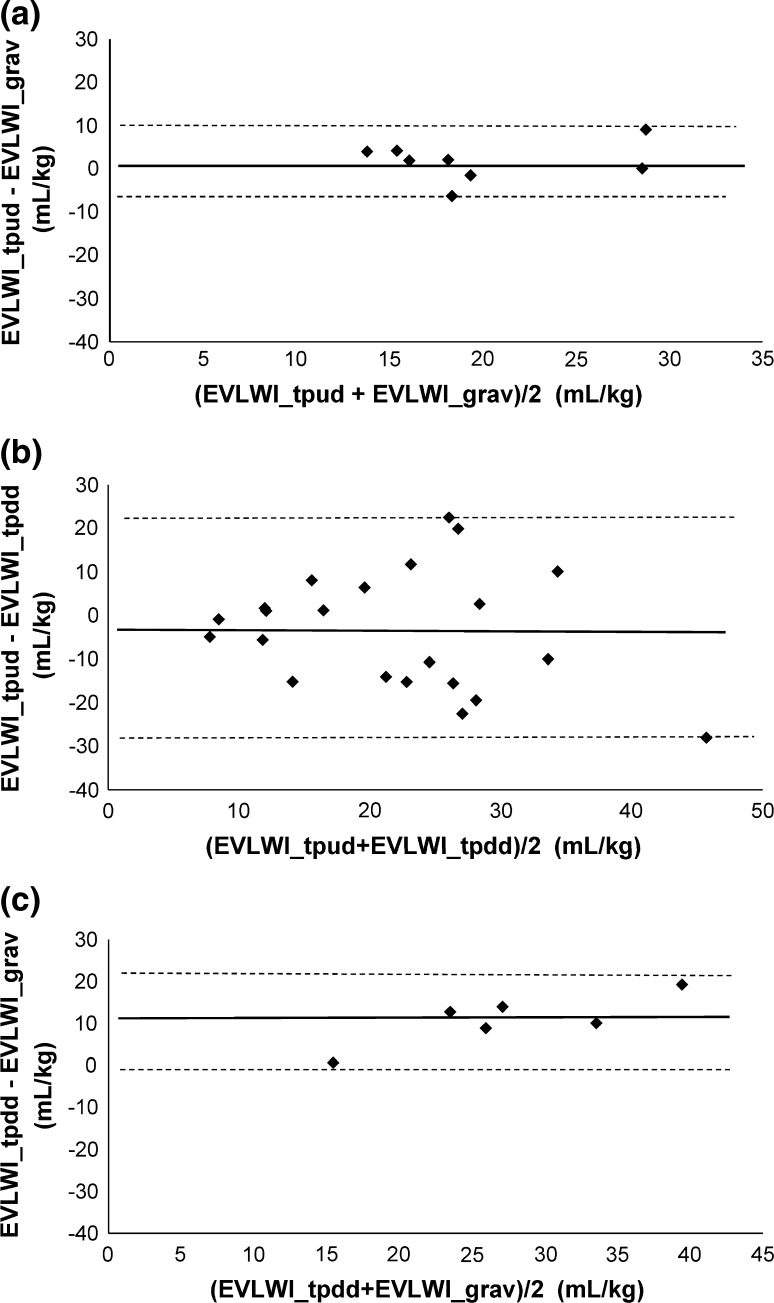
Bland–Altman plots for the comparison between **a** extravascular lung water index measured by transpulmonary ultrasound dilution (EVLWI_tpud_) and gravimetry (EVLWI_grav_), **b** EVLWI_tpud_ versus transpulmonary double indicator dilution (EVLWI_tpdd_) and **c** EVLWI_tpdd_ versus EVLWI_grav_. The *bold horizontal lines* represent the mean bias; the *dashed horizontal lines* represent the upper and lower limits of agreement

**Fig. 3:**
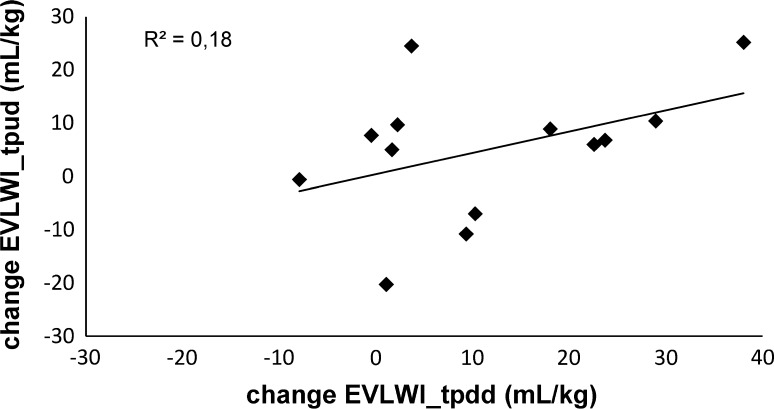
Correlation between the change in extravascular lung water index measured by transpulmonary ultrasound dilution (EVLWI_tpud_) and transpulmonary double indicator dilution (EVLWI_tpdd_) (Spearman’s rank correlation). The *continuous line* represents the linear regression line with regression coefficient r^2^

Thirteen lambs with a mean postnatal age of 15 ± 6 days (range 4–21 days) and a mean weight of 8.3 kg (range 4.1–12.3 kg) were studied. The characteristics and results of the EVLWI measurements of the lambs are shown in Table 1. EVLW could not be measured in one lamb due to technical problems (lamb 2). Four lambs (lambs 4, 7, 9 and 10) died during or after a lavage session before reliable final EVLWI_tpud_ measurements could be performed, leaving eight lambs to compare EVLWI_tpud_ with EVLWI_grav_. Mean EVLWI_grav_ was 19.0 mL/kg (SD 5.3 mL/kg). As the COLD device was out of order during the last three experiments, 9 lambs—and a total of 22 measurements—were eligible for comparison of EVLWI_tpud_ and EVLWI_tpdd_. Comparison between EVLWI_tpdd_ and EVLWI_grav_ was possible in six animals (1, 3, 5, 6, 8, 10). In the lambs who were subjected to lung lavages, the initial mean EVLWtpud was 13.3 mL/kg (SD 8.0 mL/kg) which increased after multiple lung lavages to a final mean EVLWI_tpud_ of 23 mL/kg (SD 6.3 mL/kg). This increase in lung water was accompanied by a significant increase in oxygenation index (*p* < 0.001) and PEEP levels (*p* < 0.001). Figure 1 shows the results of the EVLWI measured by TPUD (a) and TPDD (b) during the experiment and visualizes the final EVLWI measurements of the different methods (c). Bland–Altman plots for the differences between (a) EVLWI_tpud_ and EVLWI_grav_, (b) EVLWI_tpud_ and EVLWI_tpdd_ and (c) EVLWI_tpdd_ and EVLWI_grav_ are illustrated in Fig. 2. The mean bias between (a) EVLWI_tpud_ and EVLWI_grav_ was 1.7 mL/kg (LOA ± 8.3 mL/kg), (b) EVLWI_tpud_ and EVLWI_tpdd_ was −3.4 mL/kg (LOA ± 25.8 mL/kg) and (c) EVLWI_tpdd_ and EVLWI_grav_ was 11.0 mL/kg (LOA ± 11.1 mL/kg). The percentage errors were 43, 109 and 59 %, respectively. Table 2 gives an overview of the studies comparing EVLWI measured by (double) dilution techniques and gravimetry. The bias between EVLWI_tpud_ and EVLWI_grav_ was not correlated with final OI (r^2^ = 0.10, *p* = 0.54), mean EVLWI (r^2^ = 0.04, *p* = 0.70) or cardiac output (r^2^ = 0.00, *p* = 0.99). There was a weak correlation with the final PEEP (r^2^ = 0.36, *p* = 0.11). The bias between EVLWI_tpud_ and EVLWI_tpdd_ was not related to the mean EVLWI (r^2^ = 0.02, *p* = 0.53) but there was a weak correlation with the PEEP (r^2^ = 0.26, *p* = 0.01), OI (r^2^ = 0.24, *p* = 0.02) and cardiac output (r^2^ = 0.28, *p* = 0.01). Figure 3 shows the correlation between changes in EVLWI measured by TPUP and TPDD (r^2^ = 0.22, *p* = 0.11).

**Table 1:** Characteristics of the lambs

Lamb	Weight (kg)	Age (days)	Total lavage (mL/kg)	CO_tpud_ (mL/kg/min) initial–end	EVLWI_tpud_ (mL/kg) initial	EVLWI_tpud_ (mL/kg) end	EVLWI_tpdd_ (mL/kg) initial	EVLWI_tpdd_ (mL/kg) end	EVLWI_grav_ (mL/kg)	Final PEEP (mmHg)	Final OI
1	8.1	21	125	221–243	12.8	17.0	11.1	15.8	15.1	7	19.7
2	7.4	21	303	254–66	NA	NA	12.3	NA	19.4	18	21.2
3	4.1	7	293	257–215	28.6	28.6	17.2	38.6	28.5	10	50.3
4	7.4	14	68	279–460	19.6	NA	11.5	NA	18.8	10	25.0
5	10.2	17	294	177–246	5.3	19.2	10.2	29.9	17.1	20	45.6
6	9.4	18	191	217–380	12.6	18.6	11.5	34.1	20.1	20	54.0
7	7.1	14	135	133–151	22.8	NA	16.3	NA	16.5	16	27.7
8	8.0	15	420	165–120	9.0	15.2	14.6	30.4	21.5	25	36.1
9	9.9	16	242	144–165	6.5	NA	21.6	NA	18.2	20	70.4
10	11.5	19	184	152–173	8.0	NA	8.8	49.1	29.8	15	52.2
11	12.3	22	98	150–117	7.0	33.3	NA	NA	24.3	15	56.8
12	5.6	4	0	373	17.5	17.5	NA	NA	13.3	5	–
13	6.7	4	0	253	15.8	15.8	NA	NA	11.8	5	–

*CO* Cardiac output, *TPUD* transpulmonary ultrasound dilution, *TPDD* transpulmonary double indicator dilution, *Grav* gravimetry, *PEEP* positive end expiratory pressure, *OI* oxygenation index, *NA* not available

**Table 2:** overview of studies comparing extravascular lung water measured by (double) dilution methods and gravimetry

Author	Method	Species	Number of measurements	Correlation	Bias (mL/kg)	LOA (mL/kg)	Mean EVLWgrav (mL/kg)	Percentage error (%)
**Double indicator dilution methods versus gravimetry**								
Pearce—1965	Isotope indicator	Dogs						
		Edema	18	–	−1.2	2.04	4.8	43
		Severe edema	5		−6.7	4.24	9.3	45
Holcroft—1978 (mL.gr EDWL)	ICG	Baboons (shock)	29	0.87	0.31	1.4	5.5	28
Mihm—1987	ICG	Humans	9	0.98	3.43	2.99	6.06	49
Rossi—2003	Molecular indicator	Pigs						
		Sham	6	0.78	−0.26	2.85	6.4	44
		Endotoxaemia	7	0.94	5.82	4.25	12.6	34
Roch—2004	ICG	Pigs						
		Oleic acid	12	0.88	−5.2	1.8	17.5	10
		HCl acid	24	0.38	−7.9	6.5	15.1	43
Maddison—2008	ICG	Pigs	10	0.55	−1.0	6.6	9.2	71
Vrancken—2015	TPUD	Lambs	8	0.74	1.7	8.3	19	43
	ICG		6	0.90	11	11.1	22	50
**Median**				**0.87**				**43**
**Single indicator dilution method versus gravimetry**								
Katzenelson—2004	TPTD	Dogs	15	0.97	3.01	2.7	17	15
Kirov—2004	TPTD	Sheep	18	0.85	4.9	5.08	8.7	37
Rossi—2006	TPTD	Pigs						
		Sham	6	–	−5.1	1.07	6.6	16
		Endotoxaemia	5	–	−5.7	4.13	11.1	37
Maddison—2008	TPTD	Pigs	10	0.43	8.5	14.5	9.5	117
Nusmeier—2014	TPTD	Lambs	9	0.93	12.2	10.2	20.2	50
**Median**				**0.87**				**37**

*LOA* limits of agreement; percentage error = 100 × [(1.96 × SD of the bias)/meanEVLWI_grav_], *ICG* indocyanin green, *EVLWgrav* extravascular lung water measured by gravimetry, *TPUD* transpulmonary ultrasound dilution, *TPTD* transpulmonary thermodilution, *EVDW* extravascular dry weight of the lungs

## Discussion

This is a validation study investigating the applicability of EVLWI measurements by the transpulmonary ultrasound dilution method. Our results show that measuring EVLWI using TPUD seems accurate when compared with the gold standard gravimetry, but the method still lacks precision. TPUD is not reliable as a monitoring device to track changes in EVLWI when compared with the TPDD.

Despite its accuracy compared to the gravimetric method, the precision of EVLWI measurements by TPUD is rather low with an error percentage of 43 %. Like TPDD, TPUD uses a double indicator technique with temperature as a diffusible indicator (which equilibrates with the extravascular space) and the isotonic saline (TPUD) as a non-diffusible indicator. EVLW is estimated by multiplying CO and the difference in mean transit time by the thermal and the non-diffusible indicator. Various factors may influence the measurement of EVLW by indicator (dye) dilution techniques [31]: the effect of high PEEP is controversial resulting in both under- or overestimation of EVLWI as an increase in PEEP can change the distribution of thermal and non-diffusable indicators by effecting pulmonary blood flow; altered lung perfusion due to (focal) lung injury leads to underestimation of EVLW and cardiac output seems to relate inversely to the difference in MTt between the non-diffusable and the thermal indicator [32, 33]; Although this study was not designed to evaluate the effects of these factors, the bias between EVLWI_tpud_ and EVLWI_grav_ was not related to the mean EVLWI, the wide range in cardiac output or the degree of lung injury (final OI) and weakly related to PEEP. The lack of precision for estimating EVLWI_tpud_ compared to gravimetric measurements might be due to several factors: (1) the limited number of data, (2) the underlying algorithm used for the calculation of EVLW is not accurate and might need some adaptation; (3) the type of lung injury model used: the surfactant wash out model with instilling and subsequently draining saline from the lung, results in a ventilation/perfusion mismatch and therefore an increase in not only the interstitial fluid, but also the intra-alveolar fluid. However, if we assume that there is a discrepancy between the intra-alveolar fluid measured by TPUD and the gravimetry (after drainage of the lungs), we would have expected an overestimation rather than both over- and underestimation of EVLWI. (4) We conducted the gravimetric measurements according to published guidelines [12]. Our gravimetric EVLWI values were slightly higher compared to those in earlier studies (see Table 2), probably due to the wash out model with an evident increase in alveolar fluid. The time-window between the last EVLWI_tpud_ and the removal of the lungs amounted to an average of 40 min. During that time, possible shifts in intra-alveolar and interstitial fluid might occur influencing the bias between the 2 methods.

To validate the possibility of tracking changes in EVLWI we compared the TPUD with the TPDD during the experiment. Overall accuracy between EVLWI_tpud_ versus EVLWI_tpdd_ was good but there were wide limits of agreement resulting in an inacceptable high percentage error. There was a trend that the disagreement between EVLWI_tpdd_ and EVLWI_tpud_ widened with higher EVLW values (Fig. 2b). Based on these comparative results, it seems that TPUD is not applicable as a EVLWI (trend) monitoring in clinical practice, also given the weak correlation between changes in EVLWI measured by these 2 methods (Fig. 3). Explanations for this lack of precision might be various. First, differences in the underlying algorithms for the calculation of EVLW by TPDD and TPUD influencing the EVLWI measurements (see Sect. 2 and “Appendices 1, 2”). EVLWI_tpud_ estimates seem to fluctuate in some of the lambs during the experiment (Fig. 1b), an observation which might be due to the inaccuracy of the TPUD measurements (and requiring adaption the algorithm) or to real fluctuations in EVLWI (as the remaining intra-alveolar fluid after incomplete saline removal after lavages might increase EVLW). On the other hand, it is known that the TPDD overestimates EVLW because temperature exchange occurs within the cardiac structures, a phenomena which becomes more apparent with increasing lung water (see also Fig. 1c). Second, one might question the reliability of the TPDD measurements as problems with the COLD device occurred in some lambs. We tried to eliminate this factor by visually inspecting each dilution curve for artefacts or other signs of inadequate measurement. The TPDD is considered the clinical gold standard to measure EVLWI based on different studies showing strong correlation between EVLWI_tpdd_ and EVLWI_grav_ [12–18, 34–36]. However, as a strong correlation does not necessary imply good agreement between two methods [28], we reviewed some previous reports and (re)calculated bias and precision if the necessary data were available (Table 2). We found that percentage errors between EVLWI_tpdd_ and gravimetric measurements varied considerably between 10 and 71 % (mean 43 %), mainly depending on the type of lung injury. In our study, the agreement between the 2 methods is rather high (percentage error 59 %, Fig. 2c), a result that might be influenced by the limited number of measurements for comparison (n = 6). Estimates of EVLWI using TPDD (in this study) should therefore be interpreted with cautions, despite the acceptance of the method as the clinical gold standard. Based on our results, it even seems that TPUD might be a better clinical tool to estimate EVLW than TPDD.

Although TPUD seems to lack some precision to estimate EVLWI in its current application, we think the method might have potential for future use in preterm and term neonates after adapting the algorithm. No bedside methods are available at this time to measure EVLWI in these patients. The diagnostic accuracy of physical examination (crackles) is poor for the detection of increased EVLW. Chest radiographic indices and lung ultrasound profiles can be used to estimate lung fluid clearance or the presence of EVLW [37, 38] but fail to give absolute values of EVLW. Moreover, in older children no correlation was found between EVLWI measured by thermodilution and a radiographic scoring system [39]. Magnetic resonance can detect lung water content in preterm infants but the method is not bedside applicable.

Our findings do need to be interpreted cautiously as they are based on a small data set due to the early loss of animals caused by respiratory instability during lavage sessions and technical problems with the TPDD device. We are aware that more data could have altered results and conclusions. Notwithstanding this limitation, we think this pilot study adds new information regarding the possibility of EVLWI measurements by TPUD, especially for (newborn) infants.

*Other limitations* We used the surfactant lavage model to induce lung edema as RDS is one of the most commonly lung injury in neonatal intensive care. However, this model might not be totally representative for other types of (neonatal) lung injury (meconium aspiration syndrome, sepsis, cardiogenic shock with increased EVLW) as it lacks some of those specific features [24]. We did not rule out any intracardiac shunts that could have interfered with EVLW estimates (both cardiac output and mean transit times are influenced by shunt circulation). On the other hand, the TPUD method is adequate in identifying shunts, and we did not detect any shunts with TPUD in the studied animals [19].

## Conclusion

EVLWI measurements using transpulmonary ultrasound dilution in a neonatal animal model shows low agreement with the clinically accepted transpulmonary double dilution method. Accuracy seems good compared to the gold standard gravimetric method but the method lacks precision in its current prototype. Based on these pilot study data, we propose that further adjustment of the algorithm is needed before the technique can be used for (neonatal) EVLWI measurements. However, larger studies are needed to support our findings.

## Appendix 1: Calculations of EVLW by transpulmonary double indicator dilution

In this double indicator dilution technique, two different indicators are injected simultaneously, using cold ICG. The cold equilibrates with the extravascular space, while the dye is rapidly bound to proteins and remains intravascular. Analysis of dilution curves yields the cardiac output (CO), the mean transit time (MTt) of the indicator, and the exponential downslope time (DSt) of the curve.

The volume of distribution for the temperature indicator corresponds to the total water content between the injection and detection sites and is referred to as the intrathoracic thermal volume (ITTV).

Multiplication of the CO by the mean transit time of the thermal indicator (MTt_ther_) yields the ITTV.

3 $${\text{ITTV}} = {\text{CO}} \times {\text{MTt}}_{\text{ther}} \;\left( {\text{mL}} \right)$$

The volume of distribution for the dye indicator which is strictly plasma bound equals the intravascular volume between the injection and detection sites and is referred to as intrathoracic blood volume (ITBV).

Multiplying the CO by the mean transit time of the strictly plasma bound ICG (MTt_ICG_) yields the ITBV:

4 $${\text{ITBV}}_{\text{TPDD}} = {\text{CO}} \times {\text{MTt}}_{\text{ICG}}$$

The difference of the thermal and blood volume is the EVLW.

5 $${\text{EVLW}} = {\text{ITTV}} - {\text{ITBV}}_{\text{TPDD.}}$$

## Appendix 2: Formulas used for the gravimetric calculation of EVLW

6 $${\text{Fw}}_{\text{s}} = \frac{{{\text{Ww}}_{\text{s}} - {\text{Wd}}_{\text{s}} }}{{{\text{Ww}}_{\text{s}} }}$$

7 $${\text{Fw}}_{\text{h}} = \frac{{{\text{Ww}}_{\text{h}} - {\text{Wd}}_{\text{h}} }}{{{\text{Ww}}_{\text{h}} }}$$

8 $${\text{Q}}_{\text{r}} = {\text{ Q}}_{\text{h}} \, \times \frac{{{\text{Hb}}_{\text{s}} }}{{{\text{Hb}}_{\text{b}} }} \times \frac{{{\text{Fw}}_{\text{h}} }}{{{\text{Fw}}_{\text{s}} }} \times {\text{Hct}}$$

9 $${\text{Q}}_{\text{b}} \, = \frac{{{\text{Q}}_{\text{r}} + \left[ {{\text{Q}}_{\text{r}} \left( {1 - {\text{Hct}}} \right)} \right]}}{\text{Hct}}$$

10 $${\text{Fw}}_{\text{b}} \, = \frac{{{\text{Ww}}_{\text{b}} - {\text{Wd}}_{\text{b}} }}{{{\text{Ww}}_{\text{b}} }}$$

11 $${\text{EVLW}} = {\text{Q}}_{\text{h}} \times {\text{Fw}}_{\text{h}} - {\text{Q}}_{\text{b}} \times {\text{Fw}}_{\text{b}} - {\text{Q}}_{\text{wt}}$$

where EVLW is extravascular lung water, Fw is fraction water, *b* is blood, *h* is homogenate, *s* is supernatant, Hct is hematocrit, H_b_ is hemoglobin concentration, Ww is wet weight, Wd is dry weight, Q is weight, Q_*wt*_ is weight of added water, Q_*b*_ is residual blood content in lungs, and Q_*r*_ is red cell mass of lung.

## References

[1] O’Brodovich H (2005). Pulmonary edema in infants and children. Curr Opin Pediatr.

[2] Zhang Z, Lu B, Ni H (2012). Prognostic value of extravascular lung water index in critically ill patients: a systematic review of the literature. J Crit Care.

[3] Jozwiak M, Silva S, Persichini R, Anguel N, Osman D, Richard C, Teboul JL, Monnet X (2013). Extravascular lung water is an independent prognostic factor in patients with acute respiratory distress syndrome. Crit Care Med.

[4] Craig TR, Duffy MJ, Shyamsundar M, McDowell C, McLaughlin B, Elborn JS, McAuley DF (2010). Extravascular lung water indexed to predicted body weight is a novel predictor of intensive care unit mortality in patients with acute lung injury. Crit Care Med.

[5] Schiffmann H, Erdlenbruch B, Singer D, Singer S, Herting E, Hoeft A, Buhre W (2002). Assessment of cardiac output, intravascular volume status, and extravascular lung water by transpulmonary indicator dilution in critically ill neonates and infants. J Cardiothorac Vasc Anesth.

[6] Kraft R, Herndon DN, Branski LK, Finnerty CC, Leonard KR, Jeschke MG (2013). Optimized fluid management improves outcomes of pediatric burn patients. J Surg Res.

[7] Lubrano R, Cecchetti C, Elli M, Tomasello C, Guido G, Di NM, Masciangelo R, Pasotti E, Barbieri MA, Bellelli E, Pirozzi N (2011). Prognostic value of extravascular lung water index in critically ill children with acute respiratory failure. Intensive Care Med.

[8] Wheeler DS, Dent CL, Manning PB, Nelson DP (2008). Factors prolonging length of stay in the cardiac intensive care unit following the arterial switch operation. Cardiol Young.

[9] Vuohelainen T, Ojala R, Virtanen A, Korhonen P, Luukkaala T, Holm P, Tammela O (2011). Decreased free water clearance is associated with worse respiratory outcomes in premature infants. PLoS ONE.

[10] Collins JC, Newman JH, Wickersham NE, Vaughn WK, Snapper JR, Harris TR, Brigham KL (1985). Relation of blood-free to blood-inclusive postmortem lung water measurements in sheep. J Appl Physiol.

[11] Julien M, Flick MR, Hoeffel JM, Murray JF (1984). Accurate reference measurement for postmortem lung water. J Appl Physiol.

[12] Pearce ML, Yamashita J, Beazell J (1965). Measurement of pulmonary edema. Circ Res.

[13] Holcroft JW, Trunkey DD, Carpenter MA (1978). Excessive fluid administration in resuscitating baboons from hemorrhagic shock, and an assessment of the thermodye technic for measuring extravascular lung water. Am J Surg.

[14] Maddison B, Giudici R, Calzia E, Wolff C, Hinds C, Radermacher P, Pearse RM (2008). Extravascular lung water volume measurement by a novel lithium-thermal indicator dilution method: comparison of three techniques to post-mortem gravimetry. Intensive Care Med.

[15] Mihm FG, Feeley TW, Jamieson SW (1987). Thermal dye double indicator dilution measurement of lung water in man: comparison with gravimetric measurements. Thorax.

[16] Katzenelson R, Perel A, Berkenstadt H, Preisman S, Kogan S, Sternik L, Segal E (2004). Accuracy of transpulmonary thermodilution versus gravimetric measurement of extravascular lung water. Crit Care Med.

[17] Kirov MY, Kuzkov VV, Kuklin VN, Waerhaug K, Bjertnaes LJ (2004). Extravascular lung water assessed by transpulmonary single thermodilution and postmortem gravimetry in sheep. Crit Care.

[18] Rossi P, Wanecek M, Rudehill A, Konrad D, Weitzberg E, Oldner A (2006). Comparison of a single indicator and gravimetric technique for estimation of extravascular lung water in endotoxemic pigs. Crit Care Med.

[19] Lindberg L, Johansson S, Perez-de-Sa V (2014). Validation of an ultrasound dilution technology for cardiac output measurement and shunt detection in infants and children. Pediatr Crit Care Med.

[20] Vrancken SL, de Boode WP, Hopman JC, Singh SK, Liem KD, van Heijst AF (2012). Cardiac output measurement with transpulmonary ultrasound dilution is feasible in the presence of a left-to-right shunt: a validation study in lambs. Br J Anaesth.

[21] Krivitski NM, Kislukhin VV, Thuramalla NV (2008). Theory and in vitro validation of a new extracorporeal arteriovenous loop approach for hemodynamic assessment in pediatric and neonatal intensive care unit patients. Pediatr Crit Care Med.

[22] Lemson J, Eijk RJ, van der Hoeven JG (2006). The “cross-talk phenomenon” in transpulmonary thermodilution is flow dependent. Intensive Care Med.

[23] Lachmann B, Robertson B, Vogel J (1980). In vivo lung lavage as an experimental model of the respiratory distress syndrome. Acta Anaesthesiol Scand.

[24] Wang HM, Bodenstein M, Markstaller K (2008). Overview of the pathology of three widely used animal models of acute lung injury. Eur Surg Res.

[25] Moser M, Kenner T (1988). Blood flow and blood volume determinations in aorta and in coronary circulation by density dilution. Basic Res Cardiol.

[26] Reuter DA, Huang C, Edrich T, Shernan SK, Eltzschig HK (2010). Cardiac output monitoring using indicator-dilution techniques: basics, limits, and perspectives. Anesth Analg.

[27] Sakka SG, Ruhl CC, Pfeiffer UJ, Beale R, McLuckie A, Reinhart K, Meier-Hellmann A (2000). Assessment of cardiac preload and extravascular lung water by single transpulmonary thermodilution. Intensive Care Med.

[28] Bland JM, Altman DG (1986). Statistical methods for assessing agreement between two methods of clinical measurement. Lancet.

[29] Critchley LA, Critchley JA (1999). A meta-analysis of studies using bias and precision statistics to compare cardiac output measurement techniques. J Clin Monit Comput.

[30] Bland JM, Altman DG (2007). Agreement between methods of measurement with multiple observations per individual. J Biopharm Stat.

[31] Michard F (2007). Bedside assessment of extravascular lung water by dilution methods: temptations and pitfalls. Crit Care Med.

[32] Carlile PV, Beckett RC, Gray BA (1986). Relationship between co and transit times for dye and thermal indicators in central circulation. J Appl Physiol.

[33] Hill SL, Elings VB, Lewis F (1981). Effect of cardiac output on extravascular lung water. Am Surg.

[34] Nusmeier A, Vrancken S, de Boode WP, van der Hoeven JG, Lemson J (2014). Validation of extravascular lung water measurement by transpulmonary thermodilution in a pediatric animal model. Pediatr Crit Care Med.

[35] Roch A, Michelet P, Lambert D, Delliaux S, Saby C, Perrin G, Ghez O, Bregeon F, Thomas P, Carpentier JP, Papazian L, Auffray JP (2004). Accuracy of the double indicator method for measurement of extravascular lung water depends on the type of acute lung injury. Crit Care Med.

[36] Rossi P, Oldner A, Wanecek M, Leksell LG, Rudehill A, Konrad D, Weitzberg E (2003). Comparison of gravimetric and a double-indicator dilution technique for assessment of extra-vascular lung water in endotoxaemia. Intensive Care Med.

[37] Raimondi F, Migliaro F, Sodano A, Umbaldo A, Romano A, Vallone G, Capasso L (2012). Can neonatal lung ultrasound monitor fluid clearance and predict the need of respiratory support?. Crit Care.

[38] Seghaye MC, Grabitz RG, Duchateau J, Busse S, Dabritz S, Koch D, Alzen G, Hornchen H, Messmer BJ, Von Bernuth G (1996). Inflammatory reaction and capillary leak syndrome related to cardiopulmonary bypass in neonates undergoing cardiac operations. J Thorac Cardiovasc Surg.

[39] Lemson J, van Die LE, Hemelaar AE, van der Hoeven JG (2010). Extravascular lung water index measurement in critically ill children does not correlate with a chest x-ray score of pulmonary edema. Crit Care.

